# Hepatic adaptations to maintain metabolic homeostasis in response to fasting and refeeding in mice

**DOI:** 10.1186/s12986-016-0122-x

**Published:** 2016-09-26

**Authors:** C. E. Geisler, C. Hepler, M. R. Higgins, B. J. Renquist

**Affiliations:** 1 School of Animal and Comparative Biomedical Sciences, University of Arizona, 4101 North Campbell Avenue, Tucson, AZ 85719 USA; 2 Department of Internal Medicine, University of Texas Southwestern Medical Center, Dallas, TX 75235 USA

**Keywords:** Ketogenesis, Gluconeogenesis, Lipolysis, Fasting, Hepatic lipid accumulation

## Abstract

**Background:**

The increased incidence of obesity and associated metabolic diseases has driven research focused on genetically or pharmacologically alleviating metabolic dysfunction. These studies employ a range of fasting-refeeding models including 4–24 h fasts, “overnight” fasts, or meal feeding. Still, we lack literature that describes the physiologically relevant adaptations that accompany changes in the duration of fasting and re-feeding. Since the liver is central to whole body metabolic homeostasis, we investigated the timing of the fast-induced shift toward glycogenolysis, gluconeogenesis, and ketogenesis and the meal-induced switch toward glycogenesis and away from ketogenesis.

**Methods:**

Twelve to fourteen week old male C57BL/6J mice were fasted for 0, 4, 8, 12, or 16 h and sacrificed 4 h after lights on. In a second study, designed to understand the response to a meal, we gave fasted mice access to feed for 1 or 2 h before sacrifice. We analyzed the data using mixed model analysis of variance.

**Results:**

Fasting initiated robust metabolic shifts, evidenced by changes in serum glucose, non-esterified fatty acids (NEFAs), triacylglycerol, and β-OH butyrate, as well as, liver triacylglycerol, non-esterified fatty acid, and glycogen content. Glycogenolysis is the primary source to maintain serum glucose during the first 8 h of fasting, while de novo gluconeogenesis is the primary source thereafter. The increase in serum β-OH butyrate results from increased enzymatic capacity for fatty acid flux through β-oxidation and shunting of acetyl-CoA toward ketone body synthesis (increased CPT1 (Carnitine Palmitoyltransferase 1) and HMGCS2 (3-Hydroxy-3-Methylglutaryl-CoA Synthase 2) expression, respectively). In opposition to the relatively slow metabolic adaptation to fasting, feeding of a meal results in rapid metabolic changes including full depression of serum β-OH butyrate and NEFAs within an hour.

**Conclusions:**

Herein, we provide a detailed description of timing of the metabolic adaptations in response to fasting and re-feeding to inform study design in experiments of metabolic homeostasis. Since fasting and obesity are both characterized by elevated adipose tissue lipolysis, hepatic lipid accumulation, ketogenesis, and gluconeogenesis, understanding the drivers behind the metabolic shift from the fasted to the fed state may provide targets to limit aberrant gluconeogenesis and ketogenesis in obesity.

## Background

The 22.9 % incidence of metabolic syndrome has driven research focused on genetic, pharmacologic, stress, and diet induced changes in metabolic status [1–4]. Interpretation and design of these studies requires an in depth understanding of the acute changes in metabolite flux induced by either fasting or meal consumption, yet the literature lacks studies that evaluate the timing of metabolic adaptations to acute changes in food consumption. Fasting durations range from 4 h to 48 h. The commonly reported “overnight fast” is indicative of the disregard for shifting physiological adaptations that accompany fasts of varying duration. Most importantly the food deprivation and resulting metabolic perturbations are often imposed without an explanation of physiological considerations.

The liver is the central tissue maintaining metabolic homeostasis as the animal shifts between fed and fasted states. Fasting induces hepatic glucose and ketone body production, glycogen depletion, and triacylglycerol accumulation [5–7]. The triacylglycerol accumulation is a response to increased adipose tissue lipolysis [7], while many of the metabolic adaptations are adaptive to prevent hepatic lipotoxicity [6, 8, 9]. Feeding a carbohydrate containing meal stimulates hepatic glucose uptake and glycolysis, repletes glycogen stores, and induces fatty acid synthesis, while inhibiting β-oxidation and ketogenesis [10–13]. Understanding the elasticity of hepatic metabolite flux and the central role of the liver in providing nutrients to peripheral tissues is essential to studies of metabolic perturbation.

We conducted two sets of studies to delineate the timing of hepatic metabolic adaptations that maintain homeostasis through the transitions from the fed to the fasted and back to the fed state across physiologically relevant durations in the mouse. The first set of studies set out to understand the duration of fast that induces glycogenolysis, gluconeogenesis, β-oxidation and ketogenesis. To assess this, we have measured serum metabolites, hepatic glycogen and triglyceride content, activity of rate limiting enzymes in gluconeogenesis and ketogenesis, and the mRNA expression of enzymes and nuclear hormone receptors that regulate flux through β-oxidation, ketogenesis, and gluconeogenesis. Subsequently, we examined the effects of re-feeding after a fast to understand the hepatic transition from the fasted to the fed state. Together these studies define the timing of changes in hepatic metabolism and aim to encourage informed application of these dietary manipulations to study metabolic diseases.

## Methods

### Animals

Twelve to fourteen week old male C57BL/6J mice were purchased from The Jackson Laboratory (Bar Harbor, ME). Individually housed mice were exposed to a 14-h light/10-h dark cycle, given ad libitum access to NIH-31 chow (Harlan Laboratories, Indianapolis, IN) and water, and adapted to the environment for 1 week prior to study initiation. Mice were housed on wood chip bedding (Harlan Laboratories; Cat #7090 Sani-Chips) to limit any potential consumption of nutrients from bedding during the fasting period. We initiated fasting at either 4, 8, 12, or 16 h prior to sacrifice 4 h after lights on for all mice. In the fast-refeed study, mice were given access to food at 4 h after lights on for 0, 1, or 2 h after 0, 8, or 16 h of fasting. These studies were approved by the Institutional Animal Use and Care Committee at the University of Arizona.

### Sample collection and storage

We anesthetized mice with isoflurane using the bell-jar method and sacrificed mice by decapitation to collect trunk blood. The blood clotted at 4 °C overnight. To collect serum, we centrifuged the blood at 3,000 × *g* for 30 min. Serum was stored at −80 °C until analysis. Whole liver was collected, immediately frozen on dry ice, and stored at −80 °C. To obtain a homogenous liver sample, we powdered whole frozen liver using a liquid nitrogen cooled mortar and pestle.

### Serum analyses

We used colorimetric assays to analyze serum β-OH butyrate (Cat. # 700190, Cayman Chemicals, Pittsburg, PA), glucose (Cat. # G7519, Pointe Scientific Inc., Canton MI), non-esterified fatty acids (NEFA; HR Series NEFA-HR, Wako Diagnostics, Richmond, VA), and triacylglycerols (Cat# T7531, Ponte Scientific Inc., Canton, MI).

### Liver analyses

We extracted total liver mRNA with TRI Reagent® (Life Technologies, Grand Island, NY), performed reverse transcription using Verso cDNA synthesis kit (Thermo Scientific, Inc., Waltham, MA) and performed real-time PCR using SYBR 2X mastermix (Bio-Rad Laboratories, Hercules, CA) and the Biorad iQ™5 iCycler (Bio-Rad Laboratories, Hercules, CA). Prior to initiating the reverse transcription reaction, RNA was cleared of any phenol contamination using a water saturated butanol and ether method [14]. Table 1 list the primers used to analyze expression of β-actin (ACTB), D-beta-hydroxybutyrate dehydrogenase type 1 (BDH1), D-beta-hydroxybutyrate dehydrogenase type 2 (BDH2), 3-hydroxy-3-methyl glutaryl CoenzymeA synthase II (HMGCS2), peroxisome proliferator activated receptor α (PPARα), uncoupling protein 2 (UCP2), glucose 6-phosphatase (G6Pase), phosphoenolypyruvate carboxykinase (PEPCK), and carnitine palmitoyltransferase (CPT1) mRNA. Raw amplification data was imported into LinReg PCR analysis software to establish efficiency of amplification [15] and output data was converted to fold change in expression using the efficiency^-∆∆Ct^ method and with β-actin as the housekeeping gene [16].

**Table 1:** Primer sequences for real-time PCR

Target	Forward primer (5′-3′)	Reverse primer (5′-3′)	Gene ID
β-actin	TCGGTGACATCAAAGAGAAG	GATGCCACAGGATTCCATA	11461
β-OH Butyrate Dehydrogenase 1	AGGCTGTGACTCTGGATTTGGG	CTGGATGGTTCTCAGTCGGTCA	71911
β-OH Butyrate Dehydrogenase 2	AGGAGCTGGAAAGACCGAGG	TCGCAATCCAGGATGGTTCCGT	69772
3-hydroxy-3-methylglutaryl-CoA Synthase II	AGAGAGCGATGCAGGAAACTT	AAGGATGCCCACATCTTTTGG	15360
Peroxisome Proliferator Activated Receptor α	AGAGCCCCATCTGTCCTCTC	ACTGGTAGTCTTGCAAAACCAAA	19013
Uncoupling Protein 2	ATGGTTGGTTTCAAGGCCACA	CGGTATCCAGAGGGAAAGTGAT	22228
Glucose 6 Phosphatase	CGACTCGCTATCTCCAAGTGA	GTTGAACCAGTCTCCGACCA	14377
Phosphoenolpyruvate Carboxykinase	CTGCATAACGGTCTGGACTTC	CAGCAACTGCCCGTACTCC	18534
Carnitine Palmitoyltransferase I	CTCCGCCTGAGCCATGAAG	CACCAGTGATGATGCCATTCT	12894

Annealing temperature for all primer pairs was 58 °C, except HMGCS2 which was 55 °C

We used the Folch method to extract total lipid from powdered liver [17]. Extracted lipids were assayed for triacylglyerol (Cat# T7531, Ponte Scientific Inc., Canton, MI) and expressed as mg triacylglycerol/g liver. Liver NEFA content was assessed by homogenizing 10–20 mg of powdered liver tissue in 10 volumes 0.1 M phosphate buffered saline. NEFA were extracted from the tissue homogenate by vortexing with 10 volumes of 100 % ethanol for 20 min. Subsequently, NEFA were measured in 50 μl of ethanol using a commercially available non-esterified fatty acid kit (HR Series NEFA-HR, Wako Diagnostics, Richmond, VA). Dilution in ethanol rather than phosphate buffered saline did not affect the absorbance resulting from the standards, but standards were diluted in 100 % ethanol so that samples and standards were in the same diluent. Liver glycogen content was measured using a previously described colorimetric assay [18].

Using previously described enzyme activity assays that rely on the NADH to NAD^+^ ratio, we measured the enzymatic drive of acetoacetate to β-OH butyrate (BDH1 activity) and gluconeogenic potential from tricarboxylic acid (TCA) cycle intermediates (PEPCK activity) in powdered liver tissue homogenates as previously described [19, 20]. To measure liver adenosine triphosphate (ATP) content, we homogenized liver in somatic cell ATP releasing agent (Cat. FLASR, Sigma Chemical Co., St. Louis, MO) and measured ATP using an ATP Determination Kit (A22066, Molecular Probes, Eugene, OR) with luciferase read in real time on Clarity™ Luminescence Microplate Reader (BioTek Instruments, Winooski, VT). Liver cyclic adenosine monophosphate (cAMP), an integrative measure of hormone signaling within the hepatocyte, was measured in powdered liver tissue by enzyme-linked immunosorbent assay (ELISA; ADI-900-066, Enzo Life Sciences, Farmingdale, NY) and expressed per gram of tissue.

### Statistical analysis

We analyzed the effect of fasting duration on all dependent variables using the mixed model in SAS Enterprise Guide 4.3 (SAS Institute Inc., Cary, NC). Probabilities of differences between means were determined using Tukey’s adjustment for multiple comparisons. The effects of re-feeding were analyzed with a two-way ANOVA including fasting duration (0 and 16 h) and re-feeding duration (0, 1, and 2 h) and their interaction as the main effects. The probabilities of differences between means were assessed within a fasting duration and within a re-feeding duration. Accordingly, a Bonferroni correction was employed. Independent variables were identified as classification variables in both models. All raw data was plotted in Graphpad PRISM® Version 5.04 for Windows (GraphPad Software, San Diego California USA, www.graphpad.com).

## Results

### Fasting duration

**Fig. 1:**
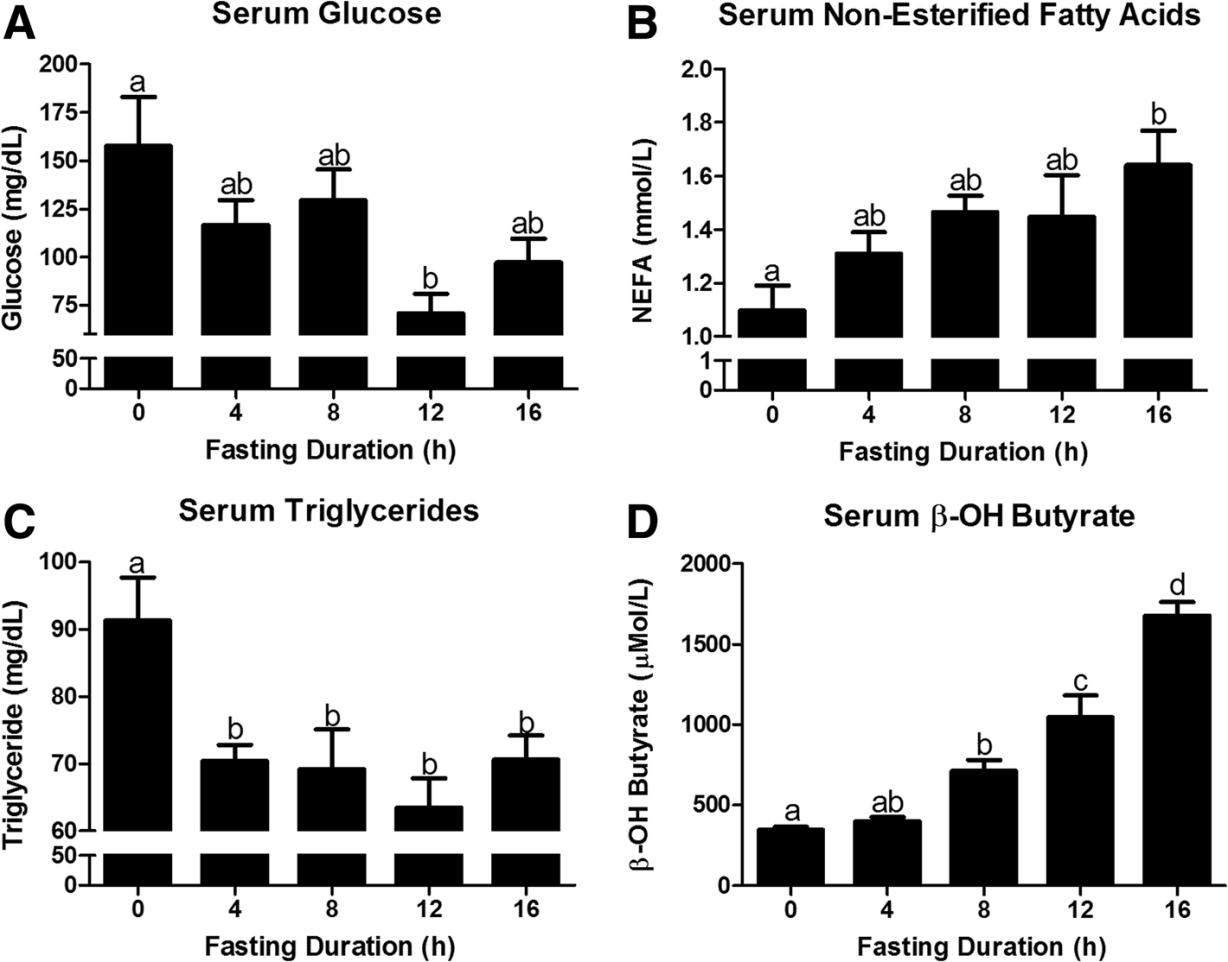
Changes in serum metabolites in response to fasting duration. Serum concentration of **a** glucose, **b** non-esterified fatty acid (NEFA), **c** triacylglycerol (TAG), and **d** β-OH Butyrate in mice that were fasted for 0, 4, 8, 12, and 16 h. ^a,b,c,d^Bars that do not share a common letter differ significantly (*P* < 0.05; *n* = 6)

Fasting decreased serum glucose levels significantly by 12 h (*P* < 0.05; Fig. 1a). In accordance with increased lipolysis at adipose tissue, serum NEFA concentrations increased with duration of fasting (*P* = 0.02; Fig. 1b). Four hours of fasting maximally decreased serum triacylglycerol concentrations (*P* < 0.05; Fig. 1c), which remained depressed with additional fasting. The most robust response to fasting was the increase in serum β-OH butyrate concentration (*P* < 0.0001; Fig. 1d). In fact, serum β-OH butyrate concentration was elevated by 8 h of fasting and continued to increase with duration of fasting. At 16 h of fasting, serum β-OH butyrate was approximately 5 times greater than baseline levels. The relatively steady serum glucose concentrations and elevation in serum β-OH butyrate during a fast result from the shift toward hepatic glucose and ketone body production.

**Fig. 2:**
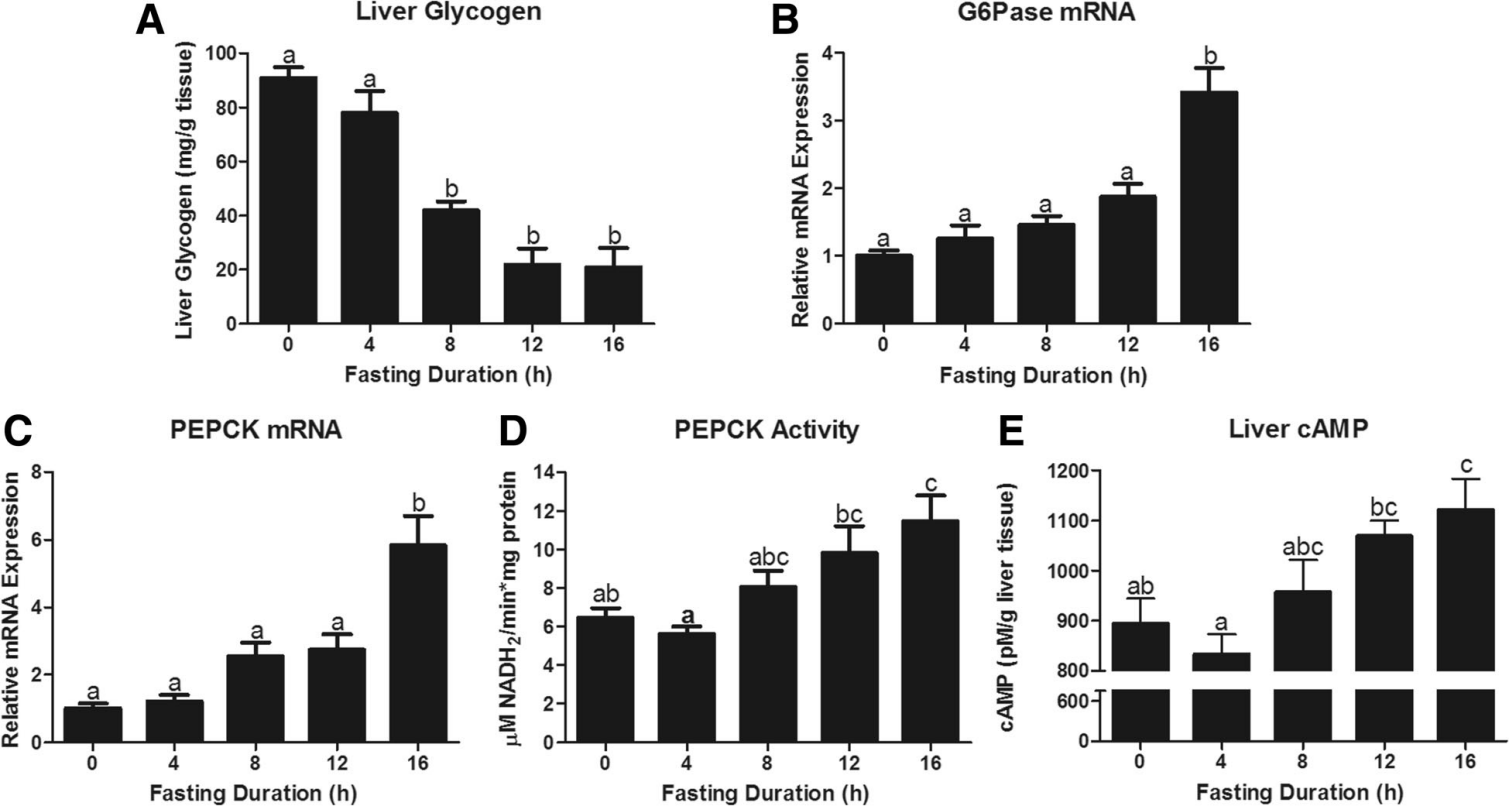
Hepatic glucoregulatory responses to fasting duration. Liver **a** glycogen (mg/g tissue) content, **b** Glucose 6 phosphatase (G6Pase) mRNA expression, **c** Phosphoenolpyruvate carboxykinase (PEPCK) mRNA expression, **d** PEPCK activity, and **e** cAMP concentration (pM/g tissue). ^a,b,c^ Bars that do not share a common letter differ significantly (*P* < 0.05; *n* = 6)

Hepatic glucose production relies on either glycogen breakdown or gluconeogenesis from glycerol, amino acids, or TCA cycle intermediates. Accordingly, we measured the decrease in hepatic glycogen content with fasting duration (Fig. 2a). Within 8 h more than 50 % of liver glycogen was depleted and at 12 h glycogen content was minimal. Hepatic glucose production from either glycogenolysis or gluconeogenesis is dependent on glucose-6-phosphatase. Glucose-6-phosphatase (G6P) mRNA expression rose with duration of fasting increasing 240 % by 16 h of fasting (*P* < 0.05; Fig. 2b). To assess the gluconeogenic potential from TCA cycle intermediates we assessed PEPCK activity and gene expression. PEPCK activity and mRNA expression increased with the duration of fast, reaching significance only at 16 h of fasting (Figs. 2c and d). Serum glucose decreased by 45 % between hours 8 and 12 of fasting, corresponding with maximal glycogen depletion at 12 h of fasting (Figs. 1a and 2a). However, serum glucose recovered by 28 % at 16 h fasting, when the greatest level of PEPCK activity was observed. This suggests a heavier reliance on gluconeogenesis to maintain serum glucose concentrations after glycogen stores have been exhausted. PEPCK mRNA and PEPCK activity are altered by cAMP, a downstream messenger increased by glucagon and decreased by insulin [21–25]. Interestingly, this integrative measure of insulin and glucagon signaling at the liver, followed a nearly identical pattern as PEPCK activity. In fact, cAMP was minimal at 4 h of fasting and increased linearly with time to 16 h (Fig. 2e). Accordingly, and the hepatic cAMP concentration and PEPCK activity were highly correlated (R^2^ = 0.47).

**Fig. 3:**
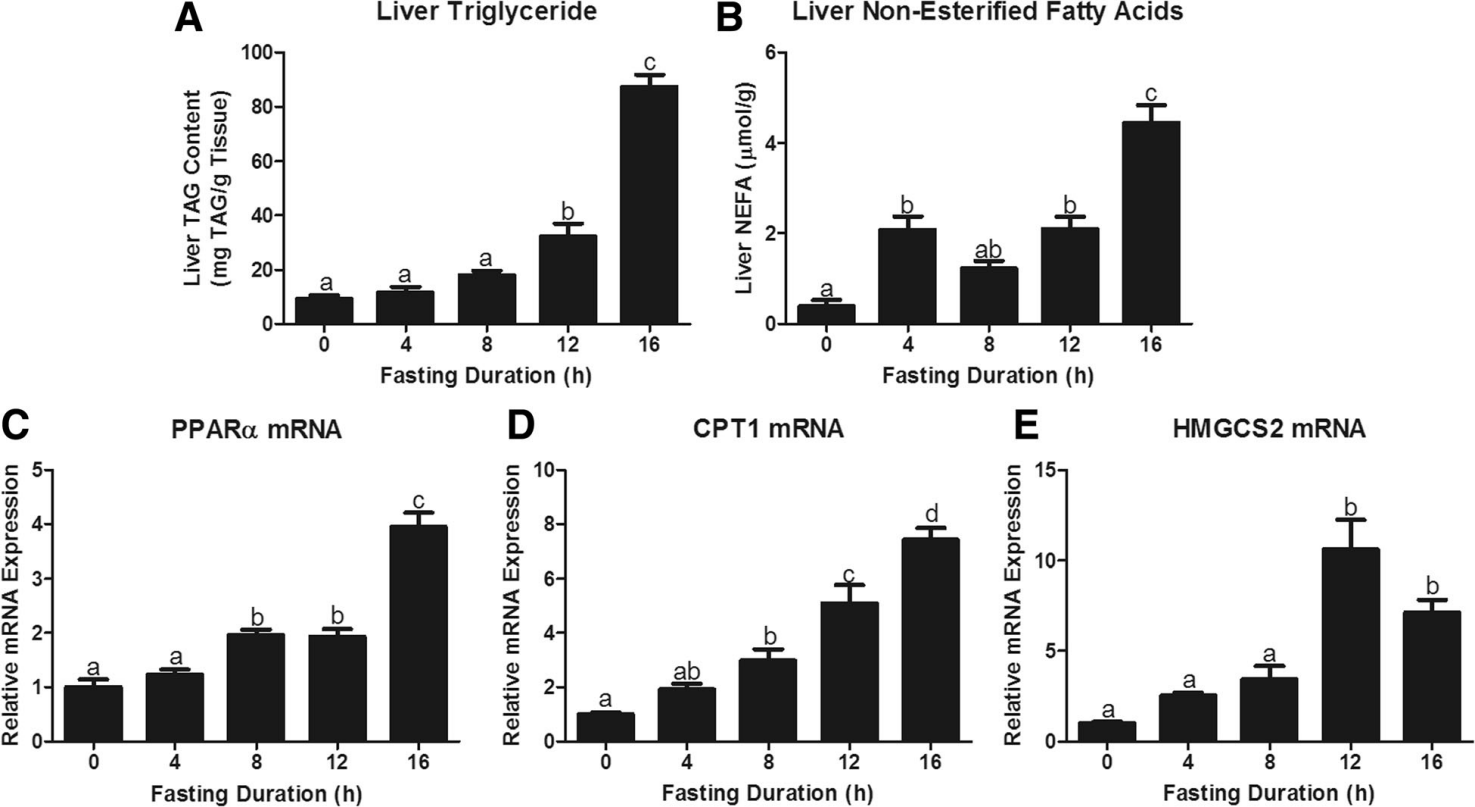
Hepatic lipid storage and metabolism responses to increasing fasting duration. Liver **a** Triacylglycerol (TAG) content, **b** Non-Esterified Fatty Acid, **c** Peroxisome proliferator-activated receptor alpha (PPARα) mRNA expression, **d** Carnitine palmitoyl transferase I (CPT1) mRNA expression, and **e** Hydroxymethylglutaryl Coenzyme A Synthase 2 (HMGCS2) mRNAexpression. ^a,b,c,d^Bars that do not share a common letter differ significantly (*P* < 0.05; *n* = 6)

The liver is the primary source of β-OH butyrate. To understand the induction of ketogenesis we first measured the hepatic accumulation of liver triacylglycerol and non-esterified fatty acids, the primary substrate fueling ketone synthesis. Liver triacylglycerol and non-esterified fatty acid concentrations increased with duration of fast (*P* < 0.0001; Fig. 3a and b). In fact, a significant rise in liver NEFA was observed within 4 h of fasting. The lipolytic and ketogenic responses to fasting depend, in part, on expression of PPARα, a NEFA activated nuclear hormone receptor, which promotes expression of genes essential to enhanced ketogenesis (CPTI, HMGCS2, BDH1, and UCP2; [8, 26–28]). Fasting increased expression of PPARα mRNA within 8 h and expression continued to increase out to 16 h (*P* < 0.05, Fig. 3c). CPT1 mRNA was also significantly elevated at 8 h and continued to rise to 16 h (*P* < 0.0001; Fig. 3d). In the fasted liver, CPT1 encourages flux of fatty acids through β-oxidation, resulting in the production of acetyl-CoA [29]. HMGCS2 is then required for the flux of acetyl-CoA into ketogenesis. Twelve and 16 h of fasting increased hepatic HMGCS2 mRNA expression (*P* < 0.0001, Fig. 3e).

**Fig. 4:**
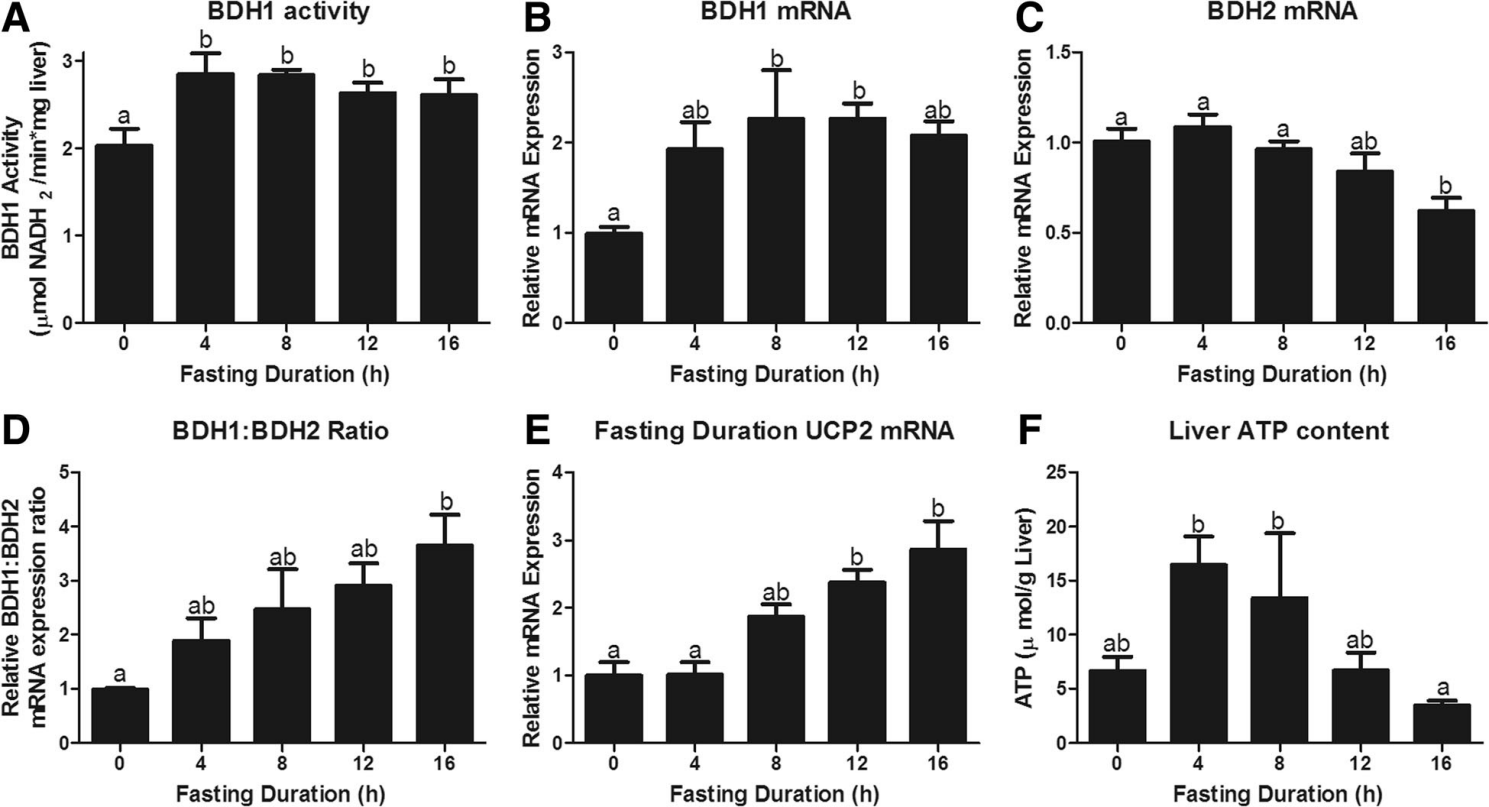
Mechanisms that regenerate NAD^+^ to allow for continued metabolic flux through β-oxidation and the tricarboxylic acid cycle. First, we present **a** β-OH butyrate dehydrogenase 1 (BDH 1) activity and **b** BDH1 mRNA expression to understand the potential regeneration of NAD^+^ as acetoacetate is converted to β-OH butyrate by β-OH butyrate dehydrogenase 1. **c** BDH2 converts β-OH butyrate to acetoacetate and in turn reduces NAD^+^ to NADH. **d** By assessing the relative ratio of BDH1:BDH2 we can see that as fasting duration is extended so is the flux from acetoacetate to β-OH butyrate which will increase the regeneration of NAD+. Finally, we shown that uncoupling protein 2 expression increases with fasting duration (**e**), leading to decreased synthesis of ATP and decreased hepatic ATP content (**f**). ^a,b^Bars that do not share a common letter differ significantly (*P* < 0.05; *n* = 6)

The flux of fatty acids through β-oxidation and acetyl-CoA through the tricarboxylic acid cycle increases hepatic mitochondrial NADH production. Without regeneration of NAD^+^, there would be limited flux of fatty acids through β-oxidation and decreased production of acetyl-CoA, which would limit ketogenesis. The liver has adapted 2 methods to regenerate NAD^+^ during a fast. First, it can increase the ratio of β-OH butyrate to acetoacetate production by altering the expression of BDH1 and BDH2. BDH1 primarily catalyzes the conversion of acetoacetate to β-OH butyrate and simultaneously NADH to NAD^+^, while BDH2 catalyzes the reverse reaction. Hepatic BDH1 activity increased within 4 h of fasting (*P* = 0.02; Fig. 4a). This preceded a significant increase in BDH1 mRNA, which was significantly elevated by fasting at 8 and 12 h (*P* < 0.05; Fig. 4b). Fasting decreased BDH2 mRNA expression significantly by 16 h (*P* < 0.05; Fig. 4c). By increasing BDH1 and decreasing BDH2, fasting increased the BDH1:BDH2 ratio to favor synthesis of β-OH butyrate and NAD^+^ (Fig. 4d). Alternatively, the liver can regenerate NAD^+^ by uncoupling electron transport and oxidative phosphorylation through upregulation of uncoupling protein 2, a PPARα responsive gene. We observe a robust fasting induced increase in UCP2 expression (*P* < 0.0001; Fig. 4e). This increase in hepatic UCP2 expression is expected to decrease hepatic ATP synthesis, explaining the reduction in hepatic ATP content following an overnight fast [30, 31]. Accordingly, liver ATP content decreased as the fasting duration went from 4 and 8 to 16 h (*P* = 0.02; Fig. 4f).

### Re-feeding after fasting

**Fig. 5:**
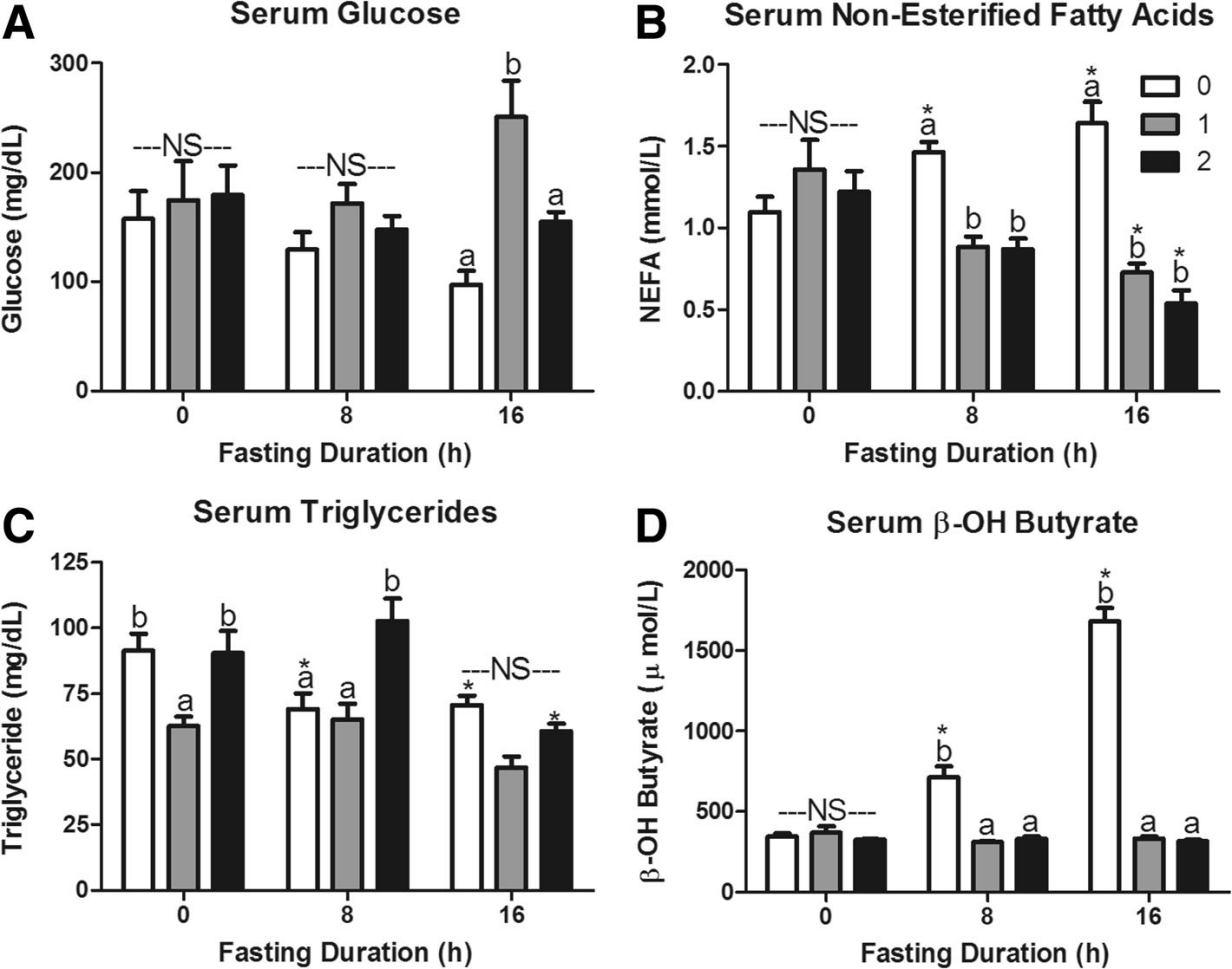
Serum metabolites in response to re-feeding. Serum **a** glucose, **b** non-esterified fatty acids (NEFA), **c** triacylglycerol (TAG), and **d** β-OH butyrate concentrations in mice fasted for 0, 8, or 16 h then allowed to re-feed for 0 (*white bars*), 1 (*grey bars*), or 2 (*black bars*) hours. ^*^Denotes a significant difference from 0 h fasting within re-feeding duration (*P* < 0.05). ^a,b^Bars that do not share a common letter differ significantly within fasting duration (*P* < 0.05; *n* = 3–6). NS, no significant differences within a fasting duration (*P* > 0.05)

**Fig. 6:**
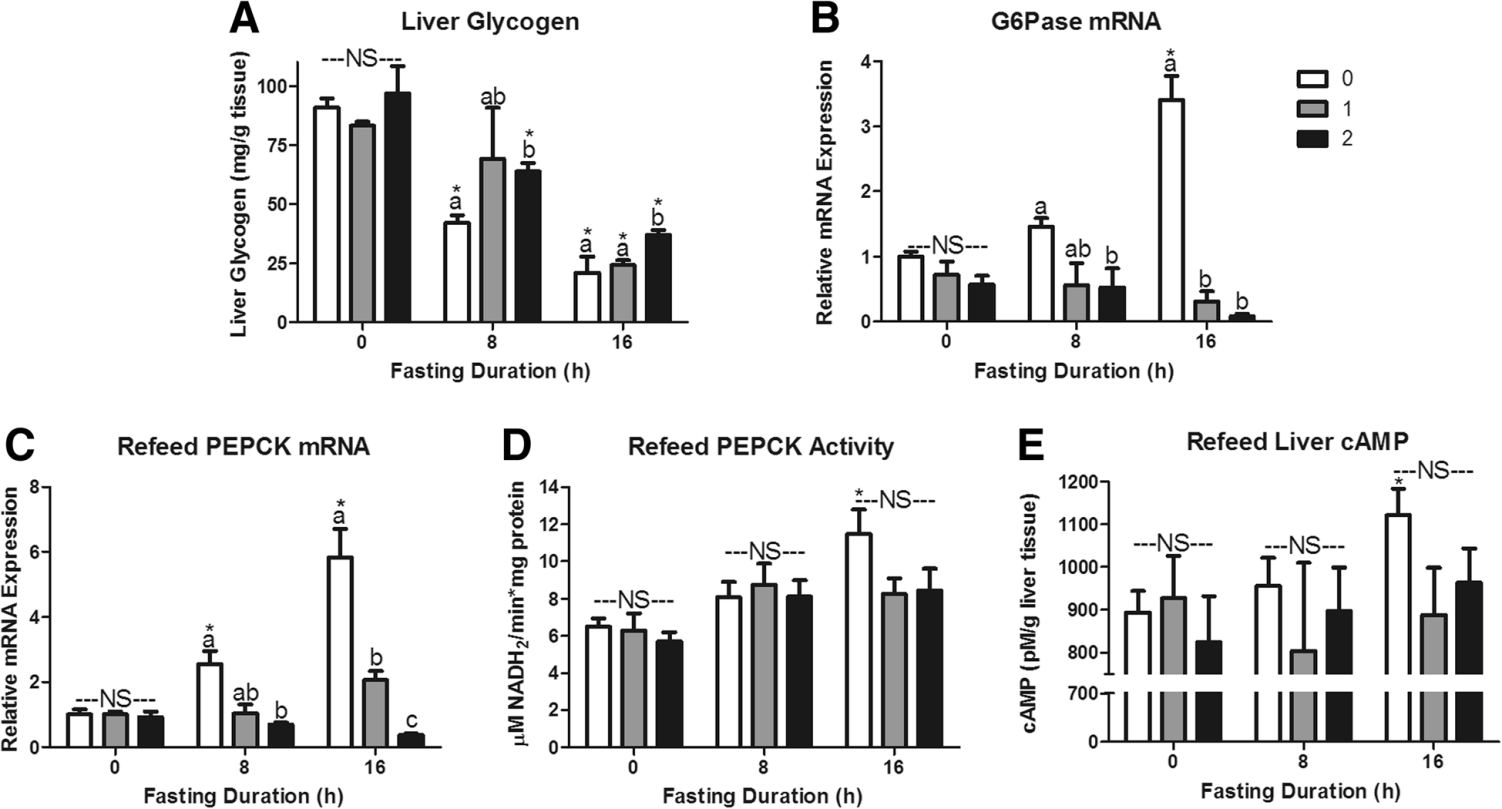
Hepatic glucoregulatory responses to re-feeding after a fast. Liver **a** glycogen (mg/g tissue) content, **b** Glucose 6 phosphatase (G6Pase) mRNA expression, **c** Phosphoenolpyruvate carboxykinase (PEPCK) mRNA expression, **d** PEPCK activity, and **e** cAMP concentration (pM/g tissue). ^*^Denotes a significant difference from 0 h fasting within re-feeding duration (*P* < 0.05). ^a,b^Bars that do not share a common letter differ significantly within fasting duration (*P* < 0.05; *n* = 3–6). NS, no significant differences within a fasting duration (*P* > 0.05)

**Fig. 7:**
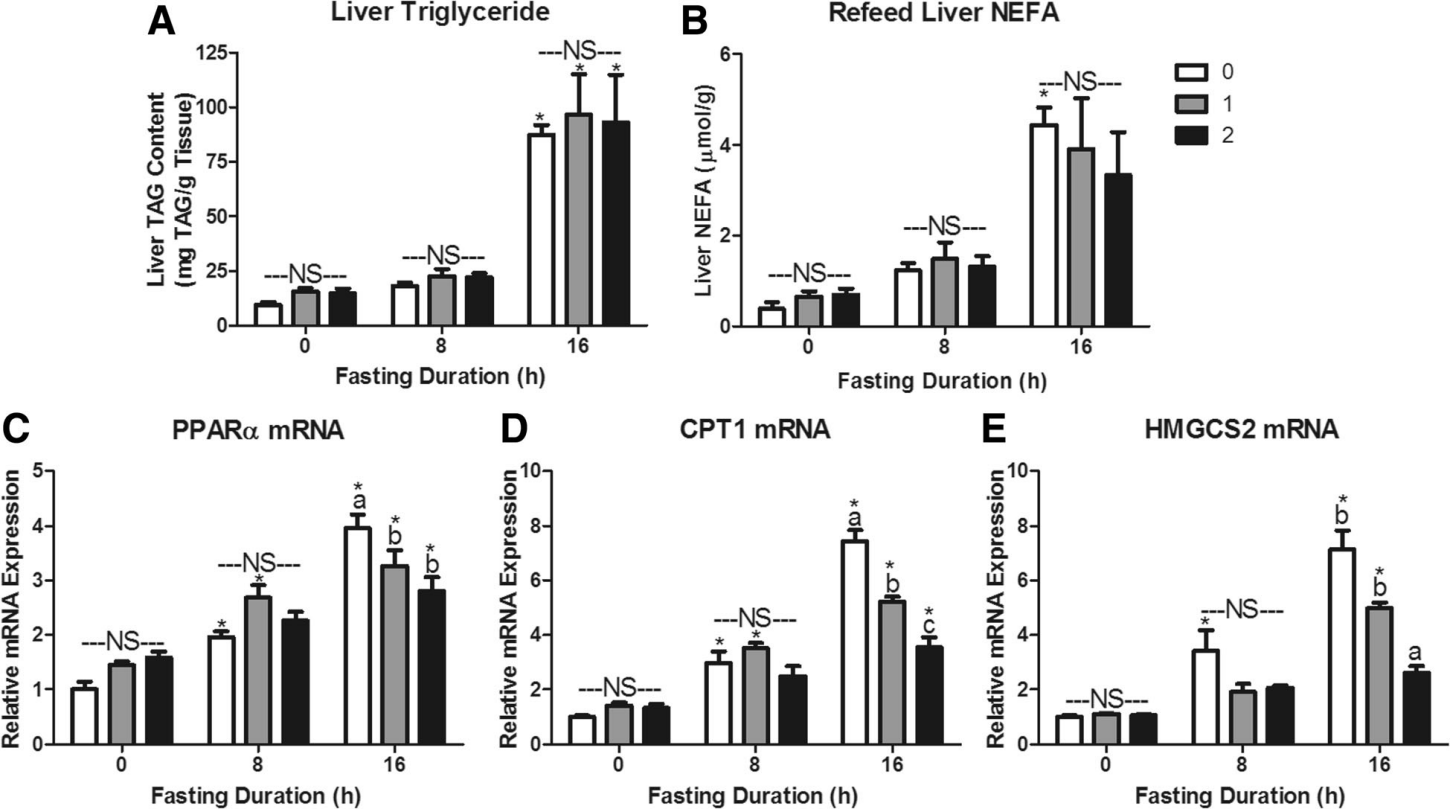
Hepatic lipid storage and metabolism responses to re-feeding after a fast. Liver **a** Triacylglycerol (TAG) content, **b** Non-Esterified Fatty Acid (NEFA) content, **c** Peroxisome proliferator-activated receptor alpha (PPARα) mRNA expression, **d** Carnitine palmitoyl transferase I (CPT1) mRNA expression, and **e** Hydroxymethylglutaryl Coenzyme A Synthase 2 (HMGCS2) mRNA expression. ^*^Denotes a significant difference from 0 h fasting within re-feeding duration (*P* < 0.05). ^a,b,c^Bars that do not share a common letter differ significantly within fasting duration (*P* < 0.05; *n* = 3–6). NS, no significant differences within a fasting duration (*P* > 0.05)

**Fig. 8:**
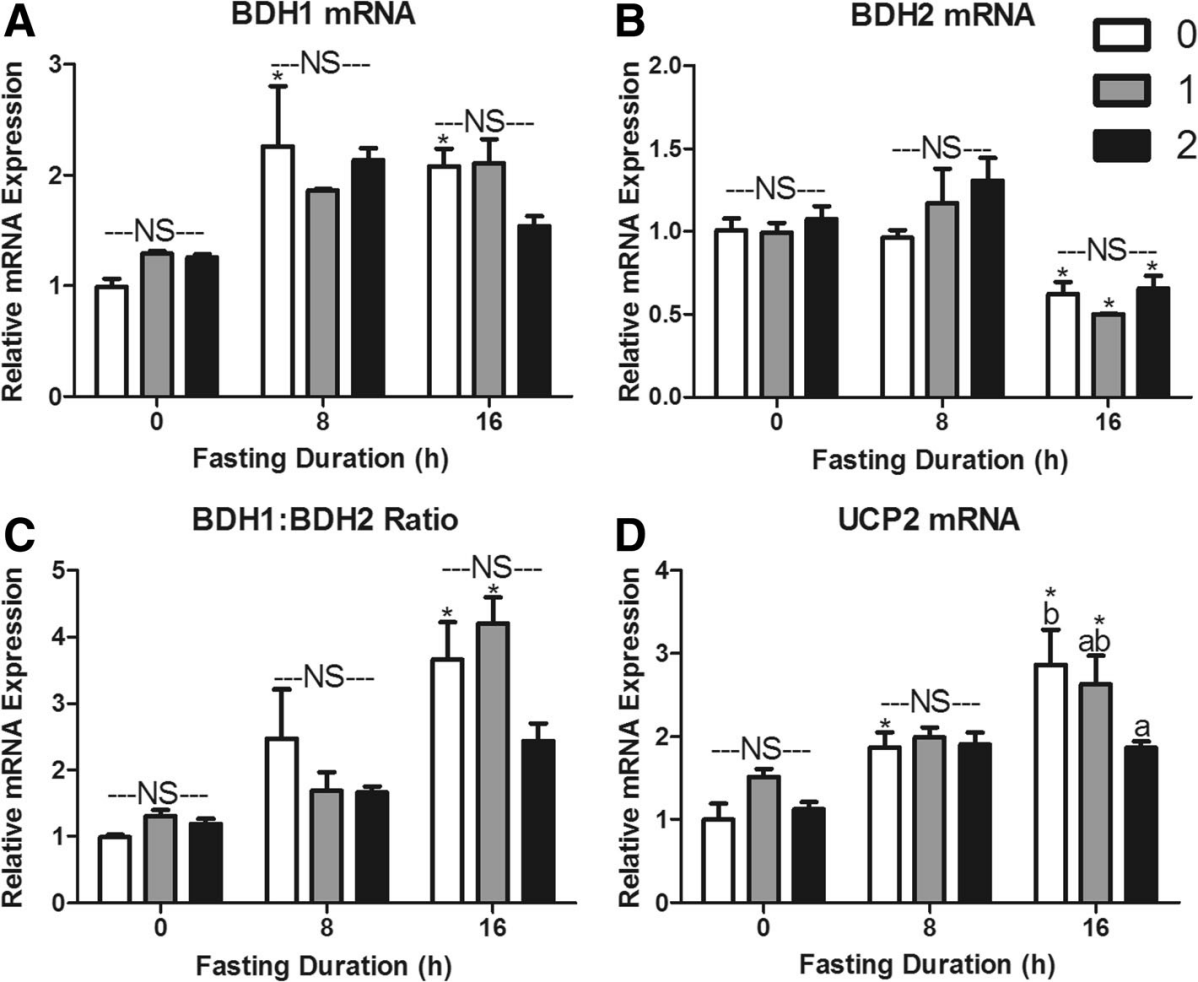
Re-feeding induced changes in hepatic **a** β-OH butyrate dehydrogenase I (BDH1), **b** β-OH butyrate dehydrogenase II (BDH2), **c** BDH1:BDH2 mRNA expression ratio, and **d** uncoupling protein 2 (UCP2) mRNA expression. ^*^Denotes a significant difference from 0 h fasting within re-feeding duration (*P* < 0.05). ^a,b^ Bars that do not share a common letter differ significantly within fasting duration (*P* < 0.05; *n* = 3–6). NS, no significant differences within a fasting duration (*P* > 0.05)

The 1 h food intakes in mice fasted for 0, 8 or 16 h were 0.10 ± 0.03, 0.60 ± 0.06 and 0.81 ± 0.05 g, respectively. The food intake during the second hour of refeeding was 0.03 ± 0.02, 0.11 ± 0.01 and 0.34 ± 0.10 g, respectively. This food intake data provides context for re-feeding responses presented in Figs. 5, 6, 7, and 8.

In mice that were maintained on ad libitum feeding throughout the study (0 h fasting duration), food was never removed, yet the time of sacrifice was 1 or 2 h later to match that in the fast-refed groups. Accordingly, there was little response to this delay in sacrifice. In fact, the only significant effect in the 0 h fasted group, was a decrease in serum triacylglycerols at 1 h of “re-feeding” that was not evident at 2 h (*P* = 0.047; Fig. 5c). All other measures of serum metabolites, hepatic mRNA expression, hepatic enzyme activity, and hepatic metabolites showed no effect of sacrificing the mice 1 or 2 h later (*P* > 0.05). Thus, responses to 1 and 2 h of refeeding observed in 8 or 16 h fasted mice are a result of the re-feeding following a fast.

The hyperphagia following a 16 h fast elevated serum glucose 1 h after re-feeding (*P* < 0.05; Fig. 5a). Re-feeding suppressed serum NEFA levels in both 8 and 16 h fasted mice (*P* < 0.001; Fig. 5b). Interestingly, 2 h of re-feeding returned serum TAG to ad libitum fed levels in 8 h fasted mice, but had no effect in 16 h fasted mice (Fig. 5c). The 8 and 16 h fasting induced increases in serum β-OH butyrate were erased within 1 h of re-feeding and remained the same as ad libitum fed mice 2 h after initiation of refeeding (Fig. 5d).

We expected the post-fast hyperphagia to increase serum glucose, resulting in hormonal changes that stimulate hepatic glycogen synthesis and inhibit hepatic glucose production. Liver glycogen concentration increased significantly with 2 h of refeeding, but did not return to concentrations seen in ad libitum fed mice (Fig. 6a). As expected, refeeding robustly depressed G6Pase mRNA expression in both 8 and 16 h fasted mice (*P* < 0.0001; Fig. 6b) [32]. Within 1 h of re-feeding hepatic PEPCK activity was no longer elevated relative to ad libitum fed mice (Fig. 6d). The response to re-feeding was more evident in PEPCK mRNA expression as 1 and 2 h of refeeding depressed 16 h fasting expression by 65 and 93 %, respectively (*P* < 0.0001; Fig. 6c).

The robust depression in serum β-OH butyrate indicates that ketogenesis is dramatically depressed within 1 h of refeeding. Substrate is not limiting, as hepatic triacylglycerol and non-esterified fatty acid content were not affected by 2 h of re-feeding in either 8 or 16 h fasted mice (*P* > 0.1; Fig. 7a and b). To understand the gene expression changes that may mediate this robust decrease in ketogenesis we measured PPARα, CPTI, HMGCS2, BDH1, and BDH2 mRNA expression changes in response to refeeding. A decrease in mRNA expression is a combined measure of decreased expression and increased mRNA turnover. After 8 h of fasting, refeeding did not significantly decrease expression of PPARα, CPTI, or HMGCS2 mRNA (*P* > 0.05; Fig. 7c-e). However, expression of these genes did not differ from the non-fasted animals after 2 h of refeeding (*P* > 0.05). In 16 h fasted mice re-feeding for 1 h significantly decreased expression of PPARα and CPT1 mRNA (*P* < 0.05). Expression of CPT1 and HMGCS2 mRNA decreased further after 2 h of food provision (*P* > 0.05).

To examine expression of genes involved in NAD^+^ regeneration, we again measured BDH1, BDH2, and UCP2 mRNA expression. Interestingly, expression of these genes does not appear to be under robust control in response to meal consumption. BDH1 and BDH2 mRNA expression and the ratio of expression didn’t change with re-feeding in either 8 or 16 h fasted mice (Fig. 8a-c). UCP2 expression was not changed by refeeding in 8 h fasted mice, but decreased with 2 h of refeeding in 16 h fasted mice (*P* < 0.05; Fig. 8d).

## Discussion

Here we show the timing of the fasting induced metabolic shift toward gluconeogenesis, glycogenolysis, β-oxidation, and ketogenesis in the mouse. Flux through each of these pathways was evaluated using biochemical indicators of pathway activity, enzyme activity assays, and measurement of mRNA expression. Gluconeogenic potential was assessed by the change in PEPCK mRNA and activity, glycogenolytic potential by the change in hepatic glycogen content, and the potential for hepatic glucose output by G6Pase mRNA [5]. Elevated mRNA expression of gluconeogenic and ketogenic enzymes translates functionally into increased flux through these metabolic pathways. In fact, mice overexpressing PEPCK mRNA, have elevated hepatic glucose output both in vivo and in vitro [5, 33], while overexpression of G6Pase mRNA results in hyperglycemia, hyperinsulinemia, and an over 50 % reduction in liver glycogen [34, 35]. Overexpression of CPT1 or HMGCS2 increases hepatic β-oxidation and ketone synthesis [36, 37], while HMGCS2 knockdown completely eliminates the fasting induced rise in serum β-OH butyrate [38]. We measured mRNA for CPT1, a fatty acid translocase whose expression controls the rate of fatty acid flux into the mitochondria and the flux through β-oxidation [39]. Serum β-OH butyrate concentration, BDH1 activity, and expression of mRNA encoding HMGCS2, BDH1, and BDH2 were measured as indicators of ketogenic potential from acetyl-CoA.

The 24 or 48 h food deprivation that is commonly employed in rodent studies appears to better model starvation than fasting [40–44]. In fact, serum NEFA concentrations are similarly elevated by 8 h of fasting in the mouse and 24 h of fasting in the human [45]. In rats, G6Pase mRNA is increased 3.5 times in 24 h fasted rats and not further increased by 48 h fasting [32], while 48 h fast increases CPT1 mRNA 7.5 times relative to the fed animal [46]. We observed similar 3.4 and 7.4 times increases in G6Pase and CPT1 mRNA, respectively, at 16 h fasting in the mouse (Figs. 2b and 3d). Thus, maximal changes in mRNA expression induced by fasting may be achieved within 16 h in mice. We conclude that 8 h fasting in the mouse represents a realistic time point for metabolic adaptations which occur early in a fast, as hepatic triglyceride accumulation and serum β-OH butyrate are minimally elevated and hepatic glycogen reserves are not yet depleted. In the mouse, 16 h of fasting, represents a complete induction of the fasting response, as hepatic triglyceride and serum β-OH butyrate concentrations are robustly elevated and hepatic glycogen stores are exhausted. Thus, refeeding after 8 h and after 16 h represents the transition from the fasted back to the fed state after a partial and complete activation of hepatic fasting metabolic adaptations, respectively.

Serum glucose homeostasis at the onset of fasting relied heavily on depletion of hepatic glycogen content, which was almost fully exhausted by 12 h of fasting (Fig. 2a). When the fasting duration exceeded 8 h, we observed increases in hepatic gluconeogenic potential (PEPCK activity and mRNA expression, Fig. 2c and d) from TCA cycle intermediates. When re-fed, the increase in hepatic glycogen combined with a decrease in G6Pase and PEPCK mRNA indicate inhibition of hepatic glucose output. Elevated serum insulin immediately stimulates hepatic glycogen synthesis upon refeeding, and glycogen content is restored to the level of fed animals by 5 h of refeeding [47, 48]. Interestingly, hepatic glycolysis remains low during the initial refeeding phase, while gluconeogenesis remains active until hepatic glycogen levels are restored [47, 48]. In fact, early glycogen repletion is a consequence of maintained hepatic gluconeogenesis [48–50]. Thus, although PEPCK and G6Pase mRNA expression decreases immediately upon termination of a fast, the switch from gluconeogenesis to glycolysis does not occur until several hours following refeeding. During early fasting hepatic glucose output is dominated by glycogenolysis and replenishment of glycogen stores through glycogenesis is prioritized upon refeeding. Therefore, hepatic glycogen appears to play a central role in maintenance of short-term glucose homeostasis during transitions between the fed and fasted state [51].

Re-feeding immediately and robustly inhibits adipose tissue lipolysis and hepatic ketogenesis as observed by changes in serum NEFA and β-OH butyrate (Fig. 5b and d). The declines in hepatic CPT1 and HMGCS2 mRNA expression parallel the decline in β-OH butyrate, yet the decline in hepatic ketogenesis is more robust and rapid than explained by changes in gene expression alone (Fig. 7d and e). The inhibition of ketogenesis is not dependent on a depression in hepatic β-oxidation, as high levels of β-oxidation and low rates of de novo lipogenesis have been reported to continue several hours into refeeding [47, 52, 53]. More likely, it results from insulin’s inhibition of HMGCS2 activity [54].

The pancreatic hormones, insulin and glucagon, are critical mediators in coordinating the systemic response to changes in nutritional status, with the liver being a primary site of action. The insulin:glucagon ratio decreases with fasting and increases upon refeeding [55, 56]. Regulation of metabolite flux, enzyme activity, and gene expression is exerted through changes in intracellular cAMP. Glucagon increases cAMP concentrations through G_αs_ signaling at its receptor while insulin decreases cAMP concentrations by enhancing phosphodiesterase activity [57]. Elevated cAMP directly upregulates transcription of PEPCK, G6Pase, CPT1, and HMGCS2 through identified cAMP response elements (CRE) in the promoter region of these genes [22, 58–60]. The observed increase in hepatic cAMP with fasting and decline upon refeeding reflect glucagon and insulin mediated control of gene transcription (Figs. 2e and 6e).

In addition to cAMP signaling through CRE, a number of hormonally regulated transcription factors control expression of gluconeogenic, β-oxidative, and ketogenic enzymes. When fasted, decreased insulin and increased glucagon result in dephosphorylation of forkhead box proteins (FoxO) and class IIa histone deacetylases (HDAC), respectively. This dephosphorylation allows nuclear translocation of these proteins and upregulation of G6Pase and PEPCK mRNA expression [61]. FoxA2, inhibited by insulin dependent phosphorylation, stimulates transcription of β-oxidative, and ketogenic enzymes [62]. This is merely a short list of insulin and glucagon regulated transcription factors meant to demonstrate the central role of glucoregulatory hormone signaling in orchestrating the hepatic mRNA transcript expression in transitions between the fed and fasted state [63].

Peroxisome proliferator activated receptor α (PPARα), a nuclear hormone receptor that is activated by non-esterified fatty acids (NEFAs), is another transcription factor central to the metabolic shift initiated by fasting [6]. Our results propose that liver NEFA concentrations are very sensitive to the initiation of a fast, as NEFA concentrations increased 5.4 times within 4 h of fasting. (Fig. 3b). NEFA activated PPARα binds to the promoter and encourages expression of target genes involved in flux through gluconeogenesis, β-oxidation, and ketogenesis [6, 64–67]. Indicative of the integral role for PPARα in the gluconeogenic response to fasting, PPARα null mice display fasting hypoglycemia [6, 64, 68]. PPARα null mice also lack the ability to properly transition to ketogenesis despite normal NEFA mobilization from adipose tissue [6, 69]. PPARα induced expression of UCP2 is equally important to support the increased hepatic lipid oxidation and ketogenesis of fasting [70]. In fact, fasting does not increase serum β-OH butyrate in the UCP2 knockout mouse [71]. By uncoupling oxidative phosphorylation from the electron transport chain, UCP2 allows unbridled oxidation of NADH to NAD^+^, increasing the pool of NAD^+^ and allowing efficient oxidation of fatty acids to acetyl CoA through β-oxidation. Thus, PPARα signaling works to limit the potential hepatotoxic effects of lipid accumulation by enhancing lipid oxidation and ketone body synthesis. PPARα null mice display a muted gluconeogenic and ketogenic response to fasting induced lipid accumulation. Like fasting, obesity is characterized by hepatic lipid accumulation, hyperketonemia, enhanced hepatic glucose production, and decreased hepatic ATP content resulting from increased expression of UCP2 [5–9, 31, 72–75]. These metabolic changes are downstream of increased PEPCK mRNA, protein, and activity, G6Pase mRNA and protein, and CPT1 and BDH1 mRNA and appear to be common to hepatic lipid accumulation [32, 76–79]. Mice fed diets high in fructose, sucrose, or fat all develop hepatic lipid accumulation and aberrantly overexpress hepatic PEPCK, G6Pase, and UCP2 mRNA [80–82]. Interestingly, PPARα null mice, which are unable to properly upregulate gluconeogenesis and ketogenesis in response to a fast, are protected from metabolic responses (hyperglycemia and hyperketonemia) common to obesity induced lipid accumulation. Thus it appears that PPARα is integral for the metabolic adaptations/maladaptations (increased ketogenesis and gluconeogenesis) in response to either fasting or obesity-induced lipid accumulation. Given that many of the metabolic pathways that are active during fasting are also active in obesity, careful consideration must be applied toward study design and data interpretation when food depriving diet- or genetically induced obese mice.

## Conclusion

There are common hepatic adaptations to lipid accumulation resulting from either fasting or obesity. Outlining how hepatic ketogenic and gluconeogenic fluxes are normally affected by fasting and feeding is essential to optimally design studies aimed at understanding aberrant metabolic flux through these pathways. These data will allow for informed design of studies aimed at understanding the response to fasting and obesity induced maladaptations in hepatic metabolism.

## Abbreviations

ACTB: Beta-actin
ANOVA: Analysis of variance
ATP: Adenosine triphosphate
BDH1: D-beta-hydroxybutyrate dehydrogenase type 1
BDH2: D-beta-hydroxybutyrate dehydrogenase type 2
cAMP: cyclic adenosine monophosphate
CPT1: Carnitine palmitoyltransferase 1
CRE: cAMP response element
DNA: Deoxyribonucleic acid
ELISA: Enzyme linked immunosorbent assay
FoxA2: Forkhead box protein A2
FoxO: Forkhead box proteins O
G6Pase: Glucose 6-phosphatase
HDAC: Histone deacetylase
HMGCS2: 3-hydroxy-3-methyl glutaryl CoenzymeA synthase II
mRNA: messenger ribonucleic acid
NAD: Nicotinamide adenine dinucleotide
NADH: Nicotinamide adenine dinucleotide + hydrogen
NEFA: Non-esterified fatty acid
PCR: Polymerase chain reaction
PEPCK: Phosphoenolypyruvate carboxykinase
PPARα: Peroxisome proliferator activated receptor α
TAG: Triacylglyceride
TCA: Tricarboxylic acid
UCP2: Uncoupling protein 2

## References

[1] Panchal SK, Brown L (2011). Rodent models for metabolic syndrome research. J Biomed Biotechnol.

[2] Boersma GJ, Salton SR, Spritzer PM, Steele CT, Carbone DL (2012). Models and mechanisms of metabolic regulation: genes, stress, and the HPA and HPG axes. Horm Metab Res.

[3] Tamashiro KL, Sakai RR, Shively CA, Karatsoreos IN, Reagan LP (2011). Chronic stress, metabolism, and metabolic syndrome. Stress.

[4] Beltran-Sanchez H, Harhay MO, Harhay MM, McElligott S (2013). Prevalence and trends of metabolic syndrome in the adult U.S. population, 1999–2010. J Am Coll Cardiol.

[5] Yoon JC, Puigserver P, Chen G, Donovan J, Wu Z, Rhee J, Adelmant G, Stafford J, Kahn CR, Granner DK (2001). Control of hepatic gluconeogenesis through the transcriptional coactivator PGC-1. Nature.

[6] Kersten S, Seydoux J, Peters JM, Gonzalez FJ, Desvergne B, Wahli W (1999). Peroxisome proliferator-activated receptor alpha mediates the adaptive response to fasting. J Clin Invest.

[7] Renquist BJ, Murphy JG, Larson EA, Olsen D, Klein RF, Ellacott KL, Cone RD (2012). Melanocortin-3 receptor regulates the normal fasting response. Proc Natl Acad Sci U S A.

[8] Cotter DG, Ercal B, d’Avignon DA, Dietzen DJ, Crawford PA (2014). Impairments of hepatic gluconeogenesis and ketogenesis in PPARalpha-deficient neonatal mice. Am J Physiol Endocrinol Metab.

[9] Rozental P, Biava C, Spencer H, Zimmerman HJ (1967). Liver morphology and function tests in obesity and during total starvation. Am J Dig Dis.

[10] Moore MC, Coate KC, Winnick JJ, An Z, Cherrington AD (2012). Regulation of hepatic glucose uptake and storage in vivo. Adv Nutr.

[11] Abumrad NN, Cherrington AD, Williams PE, Lacy WW, Rabin D (1982). Absorption and disposition of a glucose load in the conscious dog. Am J Physiol.

[12] Moore MC, Satake S, Lautz M, Soleimanpour SA, Neal DW, Smith M, Cherrington AD (2004). Nonesterified fatty acids and hepatic glucose metabolism in the conscious dog. Diabetes.

[13] Palmquist DL, Learn DB, Baker N (1977). Re-evaluation of effects of meal feeding on lipogenic activation by glucose in rats. J Nutr.

[14] Krebs S, Fischaleck M, Blum H (2009). A simple and loss-free method to remove TRIzol contaminations from minute RNA samples. Anal Biochem.

[15] Ramakers C, Ruijter JM, Deprez RH, Moorman AF (2003). Assumption-free analysis of quantitative real-time polymerase chain reaction (PCR) data. Neurosci Lett.

[16] Livak KJ, Schmittgen TD (2001). Analysis of relative gene expression data using real-time quantitative PCR and the 2(−Delta Delta C(T)) Method. Methods.

[17] Folch J, Lees M, Sloane Stanley GH (1957). A simple method for the isolation and purification of total lipides from animal tissues. J Biol Chem.

[18] Lo S, Russell JC, Taylor AW (1970). Determination of glycogen in small tissue samples. J Appl Physiol.

[19] El-Kebbaj MS, Latruffe N, Gaudemer Y (1980). Presence of an essential arginyl residue in D-beta-hydroxybutyrate dehydrogenase from mitochondrial inner membrane. Biochem Biophys Res Commun.

[20] Phillips LJ, Berry LJ (1970). Circadian rhythm of mouse liver phosphoenolpyruvate carboxykinase. Am J Physiol.

[21] Yang J, Reshef L, Cassuto H, Aleman G, Hanson RW (2009). Aspects of the control of phosphoenolpyruvate carboxykinase gene transcription. J Biol Chem.

[22] Rucktaschel AK, Granner DK, Christ B (2000). Regulation by glucagon (cAMP) and insulin of the promoter of the human phosphoenolpyruvate carboxykinase gene (cytosolic) in cultured rat hepatocytes and in human hepatoblastoma cells. Biochem J.

[23] Wicks WD, Kenney FT, Lee KL (1969). Induction of hepatic enzyme synthesis in vivo by adenosine 3′, 5’-monophosphate. J Biol Chem.

[24] Reshef L, Hanson RW (1972). The interaction of catecholamines and adrenal corticosteroids in the induction of phosphopyruvate carboxylase in rat liver and adipose tissue. Biochem J.

[25] Dhakras PS, Hajarnis S, Taylor L, Curthoys NP (2006). cAMP-dependent stabilization of phosphoenolpyruvate carboxykinase mRNA in LLC-PK1-F+ kidney cells. Am J Physiol Renal Physiol.

[26] Song S, Attia RR, Connaughton S, Niesen MI, Ness GC, Elam MB, Hori RT, Cook GA, Park EA (2010). Peroxisome proliferator activated receptor alpha (PPARalpha) and PPAR gamma coactivator (PGC-1alpha) induce carnitine palmitoyltransferase IA (CPT-1A) via independent gene elements. Mol Cell Endocrinol.

[27] Kostiuk MA, Keller BO, Berthiaume LG (2010). Palmitoylation of ketogenic enzyme HMGCS2 enhances its interaction with PPAR alpha and transcription at the Hmgcs2 PPRE. Faseb Journal.

[28] Armstrong MB, Towle HC (2001). Polyunsaturated fatty acids stimulate hepatic UCP-2 expression via a PPARalpha-mediated pathway. Am J Physiol Endocrinol Metab.

[29] Eaton S (2002). Control of mitochondrial beta-oxidation flux. Prog Lipid Res.

[30] Berglund ED, Lee-Young RS, Lustig DG, Lynes SE, Donahue EP, Camacho RC, Meredith ME, Magnuson MA, Charron MJ, Wasserman DH (2009). Hepatic energy state is regulated by glucagon receptor signaling in mice. J Clin Invest.

[31] Cheng G, Polito CC, Haines JK, Shafizadeh SF, Fiorini RN, Zhou X, Schmidt MG, Chavin KD (2003). Decrease of intracellular ATP content downregulated UCP2 expression in mouse hepatocytes. Biochem Biophys Res Commun.

[32] Mithieux G, Vidal H, Zitoun C, Bruni N, Daniele N, Minassian C (1996). Glucose-6-phosphatase mRNA and activity are increased to the same extent in kidney and liver of diabetic rats. Diabetes.

[33] Sun Y, Liu S, Ferguson S, Wang L, Klepcyk P, Yun JS, Friedman JE (2002). Phosphoenolpyruvate carboxykinase overexpression selectively attenuates insulin signaling and hepatic insulin sensitivity in transgenic mice. J Biol Chem.

[34] Seoane J, Trinh K, O’Doherty RM, Gomez-Foix AM, Lange AJ, Newgard CB, Guinovart JJ (1997). Metabolic impact of adenovirus-mediated overexpression of the glucose-6-phosphatase catalytic subunit in hepatocytes. J Biol Chem.

[35] Trinh KY, O’Doherty RM, Anderson P, Lange AJ, Newgard CB (1998). Perturbation of fuel homeostasis caused by overexpression of the glucose-6-phosphatase catalytic subunit in liver of normal rats. J Biol Chem.

[36] Monsenego J, Mansouri A, Akkaoui M, Lenoir V, Esnous C, Fauveau V, Tavernier V, Girard J, Prip-Buus C (2012). Enhancing liver mitochondrial fatty acid oxidation capacity in obese mice improves insulin sensitivity independently of hepatic steatosis. J Hepatol.

[37] Vila-Brau A, De Sousa-Coelho AL, Mayordomo C, Haro D, Marrero PF (2011). Human HMGCS2 regulates mitochondrial fatty acid oxidation and FGF21 expression in HepG2 cell line. J Biol Chem.

[38] Hepler C, Foy CE, Higgins MR, Renquist BJ (2016). The hypophagic response to heat stress is not mediated by GPR109A or peripheral beta-OH butyrate. Am J Physiol Regul Integr Comp Physiol.

[39] Spurway TD, Sherratt HA, Pogson CI, Agius L (1997). The flux control coefficient of carnitine palmitoyltransferase I on palmitate beta-oxidation in rat hepatocyte cultures. Biochem J.

[40] Adelman R, Saul RL, Ames BN (1988). Oxidative damage to DNA: relation to species metabolic rate and life span. Proc Natl Acad Sci U S A.

[41] Andrikopoulos S, Blair AR, Deluca N, Fam BC, Proietto J (2008). Evaluating the glucose tolerance test in mice. Am J Physiol Endocrinol Metab.

[42] Ayala JE, Samuel VT, Morton GJ, Obici S, Croniger CM, Shulman GI, Wasserman DH, McGuinness OP, Consortium NIHMMPC (2010). Standard operating procedures for describing and performing metabolic tests of glucose homeostasis in mice. Dis Model Mech.

[43] Galman C, Lundasen T, Kharitonenkov A, Bina HA, Eriksson M, Hafstrom I, Dahlin M, Amark P, Angelin B, Rudling M (2008). The circulating metabolic regulator FGF21 is induced by prolonged fasting and PPARalpha activation in man. Cell Metab.

[44] Jensen TL, Kiersgaard MK, Sorensen DB, Mikkelsen LF (2013). Fasting of mice: a review. Lab Anim.

[45] Browning JD, Baxter J, Satapati S, Burgess SC (2012). The effect of short-term fasting on liver and skeletal muscle lipid, glucose, and energy metabolism in healthy women and men. J Lipid Res.

[46] Park EA, Mynatt RL, Cook GA, Kashfi K (1995). Insulin regulates enzyme activity, malonyl-CoA sensitivity and mRNA abundance of hepatic carnitine palmitoyltransferase-I. Biochem J.

[47] Holness MJ, MacLennan PA, Palmer TN, Sugden MC (1988). The disposition of carbohydrate between glycogenesis, lipogenesis and oxidation in liver during the starved-to-fed transition. Biochem J.

[48] Kuwajima M, Newgard CB, Foster DW, McGarry JD (1984). Time course and significance of changes in hepatic fructose-2,6-bisphosphate levels during refeeding of fasted rats. J Clin Invest.

[49] Newgard CB, Hirsch LJ, Foster DW, McGarry JD (1983). Studies on the mechanism by which exogenous glucose is converted into liver glycogen in the rat. A direct or an indirect pathway?. J Biol Chem.

[50] Sugden MC, Watts DI, Palmer TN, Myles DD (1983). Direction of carbon flux in starvation and after refeeding: in vitro and in vivo effects of 3-mercaptopicolinate. Biochem Int.

[51] Izumida Y, Yahagi N, Takeuchi Y, Nishi M, Shikama A, Takarada A, Masuda Y, Kubota M, Matsuzaka T, Nakagawa Y (2013). Glycogen shortage during fasting triggers liver-brain-adipose neurocircuitry to facilitate fat utilization. Nat Commun.

[52] Holness MJ, French TJ, Schofield PS, Sugden MC (1987). The relationship between fat synthesis and oxidation in the liver after re-feeding and its regulation by thyroid hormone. Biochem J.

[53] Moir AM, Zammit VA (1993). Monitoring of changes in hepatic fatty acid and glycerolipid metabolism during the starved-to-fed transition in vivo. Studies on awake, unrestrained rats. Biochem J.

[54] Arias G, Asins G, Hegardt FG, Serra D (1997). The effect of fasting/refeeding and insulin treatment on the expression of the regulatory genes of ketogenesis in intestine and liver of suckling rats. Arch Biochem Biophys.

[55] Mutel E, Gautier-Stein A, Abdul-Wahed A, Amigo-Correig M, Zitoun C, Stefanutti A, Houberdon I, Tourette JA, Mithieux G, Rajas F (2011). Control of blood glucose in the absence of hepatic glucose production during prolonged fasting in mice: induction of renal and intestinal gluconeogenesis by glucagon. Diabetes.

[56] Seitz HJ, Muller MJ, Krone W, Tarnowski W (1977). Coordinate control of intermediary metabolism in rat liver by the insulin/glucagon ratio during starvation and after glucose refeeding. Regulatory significance of long-chain acyl-CoA and cyclic AMP. Arch Biochem Biophys.

[57] Benelli C, Desbuquois B, De Galle B (1986). Acute in vivo stimulation of low-Km cyclic AMP phosphodiesterase activity by insulin in rat-liver Golgi fractions. Eur J Biochem.

[58] Louet JF, Hayhurst G, Gonzalez FJ, Girard J, Decaux JF (2002). The coactivator PGC-1 is involved in the regulation of the liver carnitine palmitoyltransferase I gene expression by cAMP in combination with HNF4 alpha and cAMP-response element-binding protein (CREB). J Biol Chem.

[59] Mues C, Zhou J, Manolopoulos KN, Korsten P, Schmoll D, Klotz LO, Bornstein SR, Klein HH, Barthel A (2009). Regulation of glucose-6-phosphatase gene expression by insulin and metformin. Horm Metab Res.

[60] Nadal A, Marrero PF, Haro D (2002). Down-regulation of the mitochondrial 3-hydroxy-3-methylglutaryl-CoA synthase gene by insulin: the role of the forkhead transcription factor FKHRL1. Biochem J.

[61] Mihaylova MM, Vasquez DS, Ravnskjaer K, Denechaud PD, Yu RT, Alvarez JG, Downes M, Evans RM, Montminy M, Shaw RJ (2011). Class IIa histone deacetylases are hormone-activated regulators of FOXO and mammalian glucose homeostasis. Cell.

[62] Wolfrum C, Asilmaz E, Luca E, Friedman JM, Stoffel M (2004). Foxa2 regulates lipid metabolism and ketogenesis in the liver during fasting and in diabetes. Nature.

[63] Goldstein I, Hager GL (2015). Transcriptional and Chromatin Regulation during Fasting - The Genomic Era. Trends Endocrinol Metab.

[64] Leone TC, Weinheimer CJ, Kelly DP (1999). A critical role for the peroxisome proliferator-activated receptor alpha (PPARalpha) in the cellular fasting response: the PPARalpha-null mouse as a model of fatty acid oxidation disorders. Proc Natl Acad Sci U S A.

[65] Rodriguez JC, Gil-Gomez G, Hegardt FG, Haro D (1994). Peroxisome proliferator-activated receptor mediates induction of the mitochondrial 3-hydroxy-3-methylglutaryl-CoA synthase gene by fatty acids. J Biol Chem.

[66] Aoyama T, Peters JM, Iritani N, Nakajima T, Furihata K, Hashimoto T, Gonzalez FJ (1998). Altered constitutive expression of fatty acid-metabolizing enzymes in mice lacking the peroxisome proliferator-activated receptor alpha (PPARalpha). J Biol Chem.

[67] Im SS, Kim MY, Kwon SK, Kim TH, Bae JS, Kim H, Kim KS, Oh GT, Ahn YH (2011). Peroxisome proliferator-activated receptor {alpha} is responsible for the up-regulation of hepatic glucose-6-phosphatase gene expression in fasting and db/db Mice. J Biol Chem.

[68] Hashimoto T, Cook WS, Qi C, Yeldandi AV, Reddy JK, Rao MS (2000). Defect in peroxisome proliferator-activated receptor alpha-inducible fatty acid oxidation determines the severity of hepatic steatosis in response to fasting. J Biol Chem.

[69] Sugden MC, Bulmer K, Gibbons GF, Knight BL, Holness MJ (2002). Peroxisome-proliferator-activated receptor-alpha (PPARalpha) deficiency leads to dysregulation of hepatic lipid and carbohydrate metabolism by fatty acids and insulin. Biochem J.

[70] Pecqueur C, Bui T, Gelly C, Hauchard J, Barbot C, Bouillaud F, Ricquier D, Miroux B, Thompson CB (2008). Uncoupling protein-2 controls proliferation by promoting fatty acid oxidation and limiting glycolysis-derived pyruvate utilization. FASEB J.

[71] Sheets AR, Fulop P, Derdak Z, Kassai A, Sabo E, Mark NM, Paragh G, Wands JR, Baffy G (2008). Uncoupling protein-2 modulates the lipid metabolic response to fasting in mice. Am J Physiol Gastrointest Liver Physiol.

[72] Chavin KD, Yang S, Lin HZ, Chatham J, Chacko VP, Hoek JB, Walajtys-Rode E, Rashid A, Chen CH, Huang CC (1999). Obesity induces expression of uncoupling protein-2 in hepatocytes and promotes liver ATP depletion. J Biol Chem.

[73] Gastaldelli A, Baldi S, Pettiti M, Toschi E, Camastra S, Natali A, Landau BR, Ferrannini E (2000). Influence of obesity and type 2 diabetes on gluconeogenesis and glucose output in humans: a quantitative study. Diabetes.

[74] Serviddio G, Bellanti F, Tamborra R, Rollo T, Capitanio N, Romano AD, Sastre J, Vendemiale G, Altomare E (2008). Uncoupling protein-2 (UCP2) induces mitochondrial proton leak and increases susceptibility of non-alcoholic steatohepatitis (NASH) liver to ischaemia-reperfusion injury. Gut.

[75] Capron JP, Delamarre J, Dupas JL, Braillon A, Degott C, Quenum C (1982). Fasting in obesity: another cause of liver injury with alcoholic hyaline?. Dig Dis Sci.

[76] Caton PW, Nayuni NK, Kieswich J, Khan NQ, Yaqoob MM, Corder R (2010). Metformin suppresses hepatic gluconeogenesis through induction of SIRT1 and GCN5. J Endocrinol.

[77] Lopez-Soldado I, Zafra D, Duran J, Adrover A, Calbo J, Guinovart JJ (2015). Liver glycogen reduces food intake and attenuates obesity in a high-fat diet-fed mouse model. Diabetes.

[78] Mannisto VT, Simonen M, Hyysalo J, Soininen P, Kangas AJ, Kaminska D, Matte AK, Venesmaa S, Kakela P, Karja V (2015). Ketone body production is differentially altered in steatosis and non-alcoholic steatohepatitis in obese humans. Liver Int.

[79] Xin C, Liu J, Zhang J, Zhu D, Wang H, Xiong L, Lee Y, Ye J, Lian K, Xu C, et al. Irisin improves fatty acid oxidation and glucose utilization in type 2 diabetes by regulating the AMPK signaling pathway. Int J Obes (Lond). 2015. 10.1038/ijo.2015.199 26403433

[80] Ruiz-Ramirez A, Chavez-Salgado M, Peneda-Flores JA, Zapata E, Masso F, El-Hafidi M (2011). High-sucrose diet increases ROS generation, FFA accumulation, UCP2 level, and proton leak in liver mitochondria. Am J Physiol Endocrinol Metab.

[81] Schultz A, Barbosa-da-Silva S, Aguila MB, Mandarim-de-Lacerda CA (2015). Differences and similarities in hepatic lipogenesis, gluconeogenesis and oxidative imbalance in mice fed diets rich in fructose or sucrose. Food Funct.

[82] Schultz A, Neil D, Aguila MB, Mandarim-de-Lacerda CA (2013). Hepatic adverse effects of fructose consumption independent of overweight/obesity. Int J Mol Sci.

